# Computational Methods for Tracking, Quantitative Assessment, and Visualization of *C. elegans* Locomotory Behavior

**DOI:** 10.1371/journal.pone.0145870

**Published:** 2015-12-29

**Authors:** Kyle Moy, Weiyu Li, Huu Phuoc Tran, Valerie Simonis, Evan Story, Christopher Brandon, Jacob Furst, Daniela Raicu, Hongkyun Kim

**Affiliations:** 1 School of Computing, College of Computing and Digital Media, DePaul University, Chicago, Illinois, United States of America; 2 Department of Cell Biology and Anatomy, Chicago Medical School, Rosalind Franklin University, North Chicago, Illinois, United States of America; Nanjing University, CHINA

## Abstract

The nematode *Caenorhabditis elegans* provides a unique opportunity to interrogate the neural basis of behavior at single neuron resolution. In *C. elegans*, neural circuits that control behaviors can be formulated based on its complete neural connection map, and easily assessed by applying advanced genetic tools that allow for modulation in the activity of specific neurons. Importantly, *C. elegans* exhibits several elaborate behaviors that can be empirically quantified and analyzed, thus providing a means to assess the contribution of specific neural circuits to behavioral output. Particularly, locomotory behavior can be recorded and analyzed with computational and mathematical tools. Here, we describe a robust single worm-tracking system, which is based on the open-source Python programming language, and an analysis system, which implements path-related algorithms. Our tracking system was designed to accommodate worms that explore a large area with frequent turns and reversals at high speeds. As a proof of principle, we used our tracker to record the movements of wild-type animals that were freshly removed from abundant bacterial food, and determined how wild-type animals change locomotory behavior over a long period of time. Consistent with previous findings, we observed that wild-type animals show a transition from area-restricted local search to global search over time. Intriguingly, we found that wild-type animals initially exhibit short, random movements interrupted by infrequent long trajectories. This movement pattern often coincides with local/global search behavior, and visually resembles Lévy flight search, a search behavior conserved across species. Our mathematical analysis showed that while most of the animals exhibited Brownian walks, approximately 20% of the animals exhibited Lévy flights, indicating that *C. elegans* can use Lévy flights for efficient food search. In summary, our tracker and analysis software will help analyze the neural basis of the alteration and transition of *C. elegans* locomotory behavior in a food-deprived condition.

## Introduction

The nematode *Caenorhabditis elegans* provides many advantages for unraveling the principles underlying functional neural circuits. *C. elegans* has a simple nervous system that consists of only 302 neurons and approximately 7000 synaptic connections [1]. Furthermore, the complete anatomical annotation of its entire nervous system provides a framework for establishing specific functional maps. Additionally, many genetic tools and mutantations can be applied to modulate neural circuits, thus expediting functional mapping. For example, we can specifically activate or inactivate specific neurons using optogenetic tools [2, 3], ablate specific neurons by expressing caspase-1 [4, 5], or reduce synaptic transmission of specific neurons by expressing tetanus toxin [6, 7]. It is clear from current *C. elegans* neural circuit studies that the basic building blocks of nervous systems (modules of neural networks) are conserved across species. Thus, the knowledge gained from *C. elegans* studies will be directly applicable to more complex mammalian nervous systems.

Functional neural mapping requires monitoring of behavioral output, and several behaviors of *C. elegans* have been quantified and analyzed. These behaviors include egg laying, the pharyngeal pumping and defecation cycle, and locomotion. *C. elegans* locomotory behavior has been traditionally classified based on visual inspection by researchers. Although this classification tends to be consistent among different researchers, it is neither quantitative nor objective and poses problems when animals exhibit subtle behavioral differences. Recent developments in worm trackers, in which the movements of worms can be digitally recorded and analyzed further with computational and mathematical tools, opened doors for precise quantification of many movement parameters, including speed, acceleration, and turning [8, 9].

Although several worm trackers have been developed thus far, their shortcomings and limitations make them not suitable for all purposes [8]. For instance, some trackers are designed for recording multiple worms at the same time [10], and others are suitable for imaging of specific neurons [11–13]. It has been challenging to record the movements of single animals that are freshly removed from bacterial food, particularly over a long period of time. These animals tend to explore a large area with frequent turns and reversals, and at high speeds. This movement pattern requires the frequent adjustment of camera position along with large spatial coverage. Such requirements often lead to unreliable tracking and premature ending of the recording. Here, we developed a new stand-alone worm tracker, which is based on Python, an open source programming language, and inexpensive, commonly available hardware components, to record food-deprived animals. To recognize the worm for tracking, we implemented an image difference algorithm, in which an image frame is subtracted from a previous frame, leaving the difference image. This algorithm increases the fidelity of worm tracking by effectively removing immobile dark blobs present on the agar surface, such as salt precipitates or air bubbles. As a proof-of-principle, we successfully recorded freely moving, food-deprived animals for an extended period of time. We further analyzed their movement using algorithms newly developed by us, such as cell occupancy, step length, and locality. Wild-type animals freshly deprived of food exhibited a previously reported behavioral transition from an initial local search in a restricted area to a global search in a broad area over time [4, 14, 15]. Intriguingly, we found that this behavioral pattern often coincides with another behavioral pattern, in which animals initially displayed small, random movement steps interrupted by relatively long trajectories. This movement pattern visually resembles Lévy flights. In Lévy flights, the movement pattern is repeated across all scales such that the rank distributions of movement step lengths are best modeled by a power-law distribution, in which step length is defined as the distance between two points that are associated with directional changes. Lévy flight search is observed in the food search behavior of other species in the wild [16–18]. We analyzed the rank distribution of step lengths from wild-type worms searching for food, and found that approximately 20 percent of off-food wild-type worms indeed exhibited Lévy flights. These results indicate that, in addition to Brownian walks, *C. elegans* uses Lévy flights to locate food efficiently in a search space that lacks sensory cues.

## Materials and Methods

Our system involves both hardware and software components that allow us to acquire and process *C. elegans* locomotory behavior. The hardware components include a recording apparatus that produces video recordings by tracking single animals with a camera, and a computer for running tracking software that controls the recording apparatus. The software components include the aforementioned tracking software, as well as software for processing and analyzing the video data.

### Hardware

**Fig 1:**
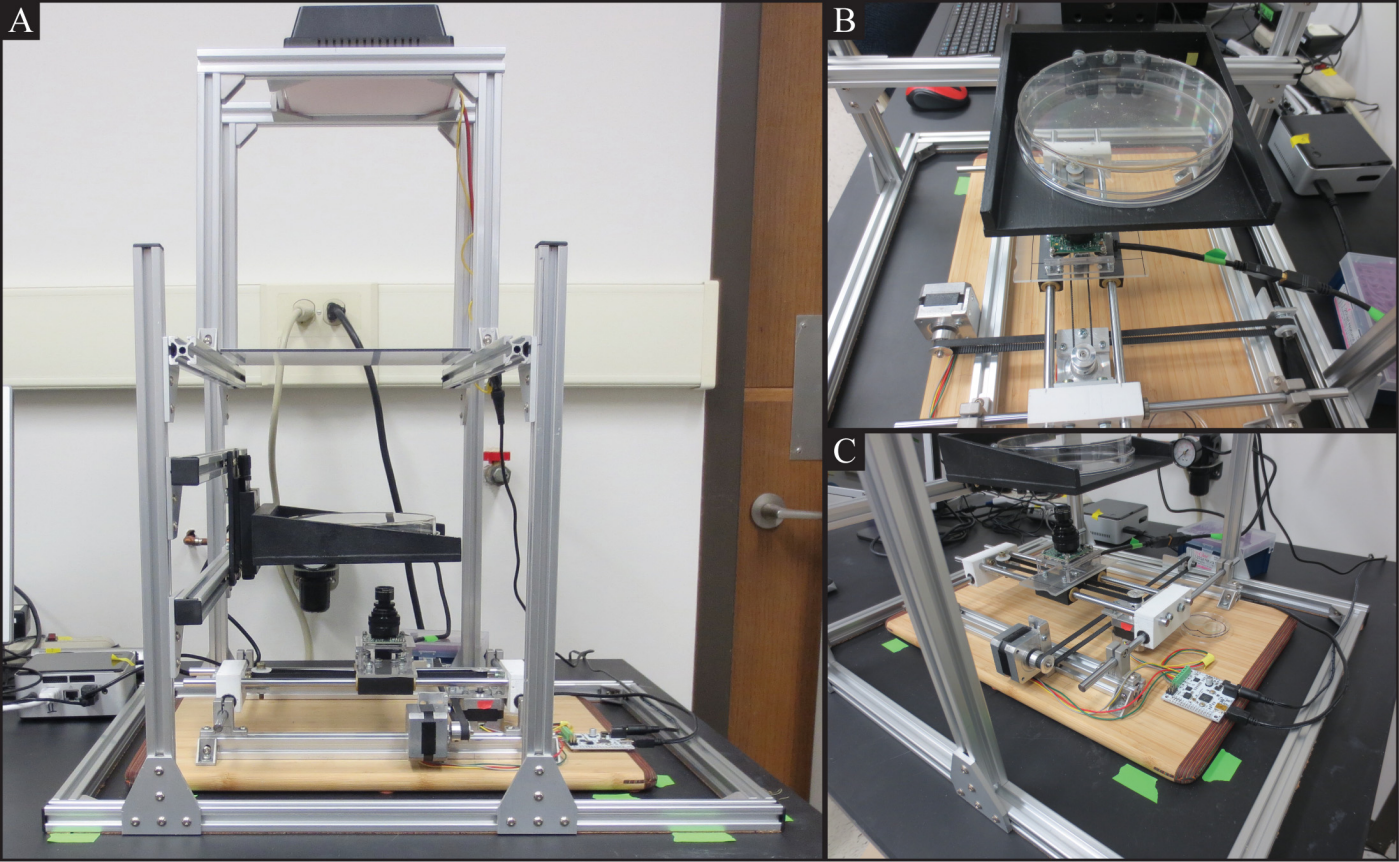
Tracker hardware. A: The tracker hardware consists of a base, the housing, and a computer. B: The housing includes a 150mm diameter agar plate on which the nematode crawls. C: The base comprises an X-Y translation stage, a camera, and two stepper motors.

The tracker (Fig 1A) consists of three parts: the base, the housing, and the computer. The components used in the tracker are common in worm labs or are otherwise relatively inexpensive, in order to minimize the cost of initial setup.

#### Base

The base of the tracker (Fig 1C) is arranged with all of the tracking device components necessary for recording the worm. It consists of an X-Y translation stage, a camera, two stepper motors, and a motor control board. The camera is mounted on the X-Y translation stage that is driven by the stepper motors. The motor control board interfaces with the tracking software, discussed later, to control the motors.

The translation stage was assembled from 8mm stainless steel rods mounted on pillow blocks (http://www.adafruit.com). The sliding components were cut from lightweight white plastic composite, and fitted with bronze sintered bearings (http://www.pbclinear.com). The motors used are NEMA 17 two phase bipolar stepper motors with a phase angle of 1.8 degrees (200 steps/rev); high precision is necessary for accurate control of the camera positioning. These are available from the Evil Mad Scientist Laboratories (EMS) for $16 each (http://www.evilmadscientist.com). The motor control board we used to interface with the PC is the EiBotBoard v1.1; a newer version, v2.0, is available from the EMS for $50. The camera is a monochrome USB 3.0 camera capable of recording at resolutions up to 1280 × 960 pixels, and at a frame rate up to 60 fps (see3CAM-10CUG from e-consystems.com, India). The camera board houses a high-resolution lens with a 25mm focal length, f5.6, with M12 mount, mounted via an M12 adapter (Edmund Optics). The lens-to-specimen distance was adjusted to give a field of view of ca. 10 mm × 7.5 mm.

#### Housing

The housing of the tracker (Fig 1A) serves as the frame that holds a NGM agar plate during recording. It is structurally independent of the base of the tracker, in order to isolate any vibration that may influence the movement of the worm. The housing, made from extruded aluminum components (http://www.adafruit.com), is designed to hold a removable 150 mm diameter NGM agar plate (Fig 1B) on an adjustable-height stage (Newport Corp.). The housing is mounted above the base of the tracker, and can be elevated to adjust the distance to the camera. Additionally, a light source (160-LED dimmable, battery-powered video light, Amazon) and diffuser are mounted above the housing, to provide adjustable lighting for recording purposes.

#### Computer

We use a dedicated Intel NUC small form factor PC ($150) running an open source Linux distribution for executing our tracking software; however, virtually any PC machine can be used. This machine connects to the motor control board and the camera via two USB ports (the computer-to-camera connection requires USB 3.0).

### Tracking and Segmentation Algorithms

#### Tracking Software

The tracking software we developed is an open source, cross-platform, standalone package written in Python that is demonstrably capable of running on low-performance machines (http://medixsrv.cstcis.cti.depaul.edu/nematodes/source/). The software uses real time input from the camera to determine the location of the worm, and adjusts the camera position to keep the worm within the field of view. All camera movements are also recorded separately from the video, which are further used to reconstruct absolute positioning.

The algorithm implemented for finding and re-centering the worm is based on a simple motion tracking mechanism: in the difference image, immobile background artifacts (such as tracks and dips) are subtracted out to black, leaving only the moving worm as a white object [19]. A single image, acquired after each camera movement, is used as a reference to subtract the background in subsequent frames. The maximum pixel intensity in the difference image provides an estimated location of the worm in that current frame.

The image subtraction method for identifying the worm position is applied at a rate of 10 Hz, subsampling from the stream being emitted by the camera. This ensures that the worm has enough time to move, and thus will not subtract itself out. To further improve the localization of the worm, a Gaussian smoothing filter is also applied. For efficiency purposes, the worm location and Gaussian filtering algorithms are applied within a cropped region always centered on the previous known worm location. The cropping mechanism is in action after 20 frames elapsed, following the program start-up and the first camera movement.

#### Worm Segmentation

**Fig 2:**
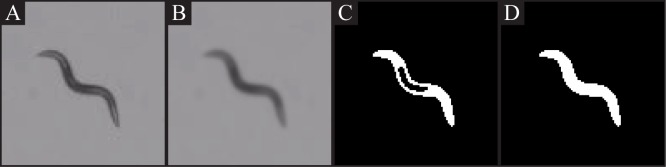
Overview of the segmentation process. A: A sample worm image. B: Gaussian filter applied on the source image. C: Thresholded image. D: Morphological closing resulting image.

The recorded videos from the tracking software are encoded as RGB AVI videos. Individual video frames are first extracted from the video and then converted to gray scale (Fig 2A). For each frame, a Gaussian filter is applied to reduce noise and homogenize the pixel intensities on the worm body, which might have a lighter intensity region due to illumination conditions (Fig 2B). As a result, the differences in the pixel intensities of the worm body are diminished and the number of segmentations with interior holes (Fig 2C) is reduced.

The filtered frames are then segmented using a dynamic thresholding algorithm, producing binary images. Each pixel is assigned a label as on-worm or off-worm based on a comparison of the pixel’s intensity to the local neighborhood average. If the ratio of the pixel intensity to the local average falls below a threshold value of 80%, the pixel is marked as potentially being a part of the worm. Pixels labeled as being part of the worm body are visually represented as completely white pixels (Fig 2D).

When holes in the worm body segmentation occur despite the preprocessing step of blurring the input image, a closing morphological operation is applied to the segmented image (Fig 2C). The closing operation first dilates the segment using a disk mask of width 5 (approximately the width of the worm), expanding the outside and inside edge of the worm and resulting in filling the holes in the worm body. Second, the excess pixels that were added to the outside of the worm are removed using erosion, which contracts the edges but does not affect the previous hole regions because the holes are fully filled and no longer have edges.

Finally, connected components are identified using a union-find data structure. The connected component with the largest area is identified as being the worm [20]. All other components are removed from the image.

#### Worm Body Centroid Determination

One feature of interest is the location of the worm as it travels during the observation period. To represent this location, we extract the centroid, or center of mass, of the worm from each frame. The centroid of the worm is determined as the average of the x and y coordinates of all M pixels on the worm body, calculated as follows:

$$\begin{array}{c} \left(C_{x},C_{y}\right)=\left(\frac{\sum_{i=1}^{M}x_{i}}{M},\frac{\sum_{i=1}^{M}y_{i}}{M}\right) \end{array}$$ (1)

#### Worm Segmentation in a Distributed Computing Environment

As the image processing algorithms are computationally expensive, processing video data of long observation periods can be a time-consuming process. In response, we have developed a distributed computing solution for utilizing a large number of workstation machines.

The segmentation process is designed for task-parallelism so that high-throughput analysis can be attained with the use of one or more machines. The software is multithreaded and can process multiple parts of the data set concurrently; this accomplishes real time performance (30 frames per second) on most individual machines. The task is further parallelized by adding multiple machines to a network, scaling performance approximately linearly with each additional machine.

This distributed network is made up of multiple machines, or nodes, that the user can control from one endpoint. The application requires a terminal node, a redistribution node, and any number of additional compute nodes. The terminal node is a machine where the user can control the network; adding or removing nodes, sending data sets to be processed, and retrieving processed data. It also acts as a scheduler for tasking the compute nodes. The redistribution node, in our case a file server with greater network bandwidth and disk performance, stores and distributes parts of the data set to the compute nodes as they require them. The compute nodes receive tasks from the terminal node and data from the redistribution node, then return processed data to the redistribution node.

### Movement and Path Analysis Algorithms

#### Movement Path Extraction

To extract the worm path on the agar plate, the worm centroid (*C*_*x*_, *C*_*y*_) is tracked as it moves throughout the video [21]. This centroid position is calculated using Eq (1), however, it is only the position in respect to the current frame. To calculate the global coordinates for the centroid, what we call a location relative to the initial camera position, we offset this centroid value by the camera’s current position coordinates in pixels. Camera position is recorded at every frame in a log file from the tracker software. The sequence of the centroids’ global coordinates represents the extracted worm path in the corresponding video.

#### Speed, Acceleration, Angle, and Angular speed

The centroids are also used to objectively quantify a worm’s movement behavior: instantaneous speed, acceleration, angle, and angular speed are calculated from the changes in centroid position between frames. Our calculations feature windowed averages with a parameter for the sample time interval *δ* that can be adjusted to balance noise reduction and data fidelity. The instantaneous speed *s* of the worm was calculated by taking the displacement between two consecutive centroid positions and dividing by the sample interval length of time [12]. The angle *φ* is calculated as the vector direction between two centroid positions with respect to the horizontal [12]. Furthermore, acceleration *a* and angular speed *ω* are the differences of consecutive values of instantaneous speed and angle, respectively. Eqs (2)–(5) provide the formulas for these movement features where *t* is time in seconds:

$$\begin{array}{c} s_{t}=\frac{\sqrt{{\left(x_{t}-x_{t-\delta }\right)}^{2}+{\left(y_{t}-y_{t-\delta }\right)}^{2}}}{\delta } \end{array}$$ (2)

$$\begin{array}{c} a_{t}=\frac{s_{t}-s_{t-\delta }}{\delta } \end{array}$$ (3)

$$\begin{array}{c} \varphi_{t}=\arctan\frac{y_{t}-y_{t-\delta }}{x_{t}-x_{t-\delta }} \end{array}$$ (4)

$$\begin{array}{c} \omega_{t}=\frac{\varphi_{t}-\varphi_{t-\delta }}{\delta } \end{array}$$ (5)

#### Cell Occupancy

Cell occupancy *O* is a metric by which the spatial search efficiency of the worm can be quantified. A greater average cell occupancy value indicates oversampling, as the worm is searching areas repeatedly, or visiting less area per unit time. Cell occupancy values are attained by dividing the physical space into a grid of square cells, and calculating the number of unique cells that the worm visits in a given time period [22]. In our implementation, we chose the grid cell size as 1 mm^2^, based on the average length of adult worms. To help understand how the worm’s search efficiency changes over, the number of unique cells visited can be counted for discrete time periods throughout the video.

#### Step Length Analysis

Rather than working with all the centroid points on the worm path, we divide the path into a series of line segments called “steps” and use the step length data to quantify the path and infer patterns in search behavior. Our algorithm is based on the work by Reynolds et al. [23] and Codling et al. [24] in which the location of a turning event was defined where “the angle between two movement segments joining three successive positional fixes is less than a critical angle”. While they applied the algorithm to understand the movement behavior of fruit flies (*Drosophila melanogaster*) from short video tracking data and to investigate optimal search strategies for foragers based on computer simulated data, we propose to apply it to quantitatively describe the movement path of *C. elegans* in the presence and absence of food for longer periods of time.

The proposed algorithm consists of three steps: path sampling, turning event identification, and step identification and length calculation.

**Fig 3:**
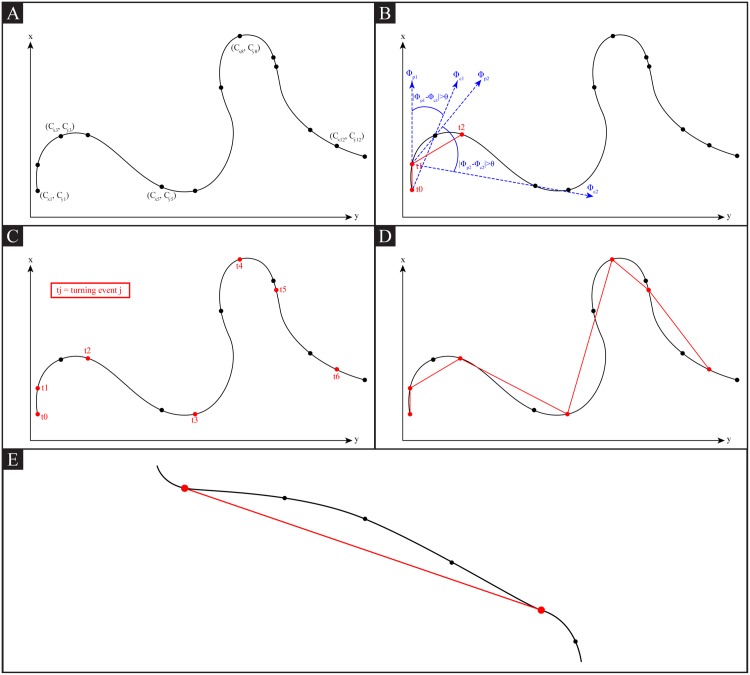
Step length analysis. A: Example of sampled centroid locations of a worm (*C*_*x*_*i*__, *C*_*y*_*i*__) at Δ*t* = 1 sec. B: Identification of “turning events”—the first two turning events are shown with their respective *φ*_*p*_*j*__ and *φ*_*c*_*j*__. C: location of all turning events. D: Resultant sampled path using steps. E: Sampled path using steps for a relatively “straight” worm path.

First, we sample the centroid coordinates of the worm (*C*_*x*_, *C*_*y*_) at a regular time interval Δ*t* = 1 sec forming a dataset of [*t*_*i*_, *C*_*x*_*i*__, *C*_*y*_*i*__] values where time *t*_*i*_ = Δ*t* ∗ *i* and *i* denotes the *i*-th point on the sampled path at a time rate of Δ*t* (Fig 3A). Second, a “turning event” (*TE*) is identified at a specific [*t*_*i*_, *C*_*x*_*i*__, *C*_*y*_*i*__] (Fig 3B and 3C) if the current movement heading, *φ*_*c*_*j*__ deviates by more than some threshold angle Θ from the heading at the previous turning event, *φ*_*p*_*j*__, where *j* denotes the index for turning events. Our current approach determines the threshold empirically as a tradeoff between the correct sampling of the path and the predictive modeling power of the generated step length data. Therefore, a new subsample of centroids is generated from the sampled path, containing only the centroid locations for the turning events [*TE*_*j*_, *C*_*x*_*j*__, *C*_*y*_*j*__]. Note that the first centroid location of the worm, [*t*_1_, *C*_*x*_1__, *C*_*y*_1__], is considered to be the initial turning event, [*TE*_0_, *C*_*x*_0__, *C*_*x*_0__]. Once the turning events are identified, a step is defined as a line segment (Fig 3D)) between two consecutive turning events, [*TE*_*j*−1_, *C*_*x*_*j*−1__, *C*_*y*_*j*−1__] and [*TE*_*j*_, *C*_*x*_*j*__, *C*_*y*_*j*__]. The length of a step *S*_*j*_ is then calculated as:

$$\begin{array}{c} S_{j}=\sqrt{{\left(C_{x_{j}}-C_{x_{j-1}}\right)}^{2}+{\left(C_{y_{j}}-C_{y_{j-1}}\right)}^{2}} \end{array}$$ (6)

Fig 3D and 3E show two hypothetical paths of *C. elegans* and their respective sampled paths using the described step length algorithm to illustrate its significance for determining patterns in the search behavior. We notice that the “curvy” path in Fig 3D resulted in a greater number of steps than the “straight” path in Fig 3E and that each of those step lengths in Fig 3D was shorter as well. We can therefore take advantage of this insight and use step length to describe various movement behaviors of the worm. A smaller mean step length over a unit of time is associated with more “curving” behavior and vice versa with a larger mean step length over time associated with more “straight” behavior.

#### Lévy Flight step length Distribution Analysis

Our initial visual assessment of the path of food-deprived wild-type animals led us to investigate the possibility of quantifying step length data using power-law distributions as described by the probability distribution formula:

$$\begin{array}{c} p\left(S_{j}\right)∼S_{j}^{-\alpha } \end{array}$$ (7)

where ‘∼’ means ‘distributed as’ and *α* is the power-law (Lévy) exponent. The Lévy exponent is constrained by the condition, 1 < *α* ≤ 3, which ensures that the distribution can be normalized with probabilities that sum to one and is characterized by a divergent variance [25]. Since in practice few empirical phenomena obey the power law for all values of *S*_*j*_, the power law applies only to values greater than some minimum *S*_*j*_; the tail of the distribution then follows a power law distribution [26]:

$$\begin{array}{c} p\left(S_{j}\right)=\frac{\alpha -1}{S_{j,min}}*{\left(\frac{S_{j}}{S_{j,min}}\right)}^{-\alpha } \end{array}$$ (8)

Assuming that the data are drawn from a distribution that follows a power law distribution for *S*_*j*_ ≥ *S*_*j*,*min*_, the scale parameter *α* can be estimated from the data using the method of maximum likelihood [26]:

$$\begin{array}{c} \hat{\alpha }=1+n{\left[\sum_{j=1}^{n}\ln\frac{S_{j}}{S_{j,min}}\right]}^{-1} \end{array}$$ (9)

where *n* is the number of points on the tail of the distribution that follow a power law distribution and *S*_*j*_ are the observed values such that *S*_*j*_ ≥ *S*_*j*,*min*_, *j* = 1‥*n*. For estimating *S*_*j*,*min*_ we use the method proposed by Clauset et al. [26] that chooses the value of *S*_*j*,*min*_ that makes the probability distribution of the measured data *p*(*S*_*j*_) and the best fit power-law model *q*(*S*_*j*_) as similar as possible above the estimator of *S*_*j*,*min*_. Using the Kolmogorov-Smirnov or KS statistic to quantify the distance between the two distributions, the estimator of *S*_*j*,*min*_ is then the value that minimizes *D* [26]:

$$\begin{array}{c} D=max_{S_{j}}\left\lceil p\left(S_{j}\right)-q\left(S_{j}\right)\right\rceil \end{array}$$ (10)

#### Locality Analysis

Since the step length data only capture whether a worm exhibits straight or curving behavior, we introduce a new metric to describe whether a worm is moving “locally” or “globally” alongside step length. To quantify this locality *L*, we use the ratio of mean speed to unique cells visited over an interval of time *τ*_*k*_ where *k* denotes the *k*th time interval *τ* from the start of the video and the mean speed *E*[*v*_*k*_] per interval *τ*_*k*_ is calculated using instantaneous speeds gathered at every frame of the video for an interval *τ*_*k*_:

$$\begin{array}{c} L\left(\tau_{k}\right)=\frac{E[v_{k}]}{O_{k}} \end{array}$$ (11)

Since every interval of time *τ*_*k*_, is the same (1 minute), *E*[*v*_*k*_] is proportional to the distance traveled by the worm over *τ*_*k*_. In other words, *D*_*k*_ = *E*[*v*_*k*_] ∗ *τ*_*k*_ for each interval *τ*_*k*_ and thus distance *D*_*k*_ is just *E*[*v*_*k*_] scaled by a constant of *τ*_*k*_. The locality metric becomes proportional to the distance:

$$\begin{array}{c} L\left(\tau_{k}\right)∼\frac{D_{k}}{O_{k}} \end{array}$$ (12)

**Fig 4:**
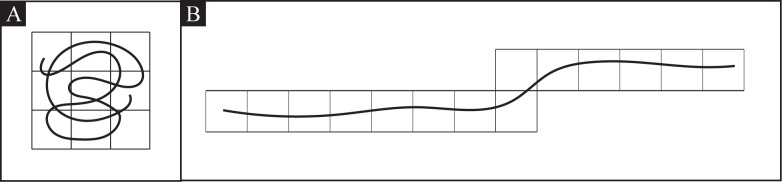
Locality movement. A: Hypothetical “local” search movement. B: Hypothetical “global” search movement.

Fig 4A and 4B show how the locality concept helps differentiate between two hypothetical local and global search movements. While the distance traveled in both cases Fig 4A and 4B is the same, the number of unique cells covered is larger for the path in Fig 4B and therefore produces a low locality for this path.

## Results

### Establishment of a New Tracker to Reliably Record *C. elegans* movement

We have developed a tracker to examine *C. elegans* food search behavior. First, we determined whether the tracker performs well, particularly in a challenging condition that causes extended movements with turns and reversals. For comparison, we observed animals under two different environments; on an agar plate seeded with E. coli (“on-food”), the nematode’s primary food source, and on an agar plate without bacterial food (“off-food”). In addition, we also determined whether *tph-1* mutants, which are defective in producing serotonin and exhibit several food-related behavioral deficits, show altered food search behavior in comparison with wild-type animals.

We have been able to observe and record on-food locomotory behavior over a few hours of time, and off-food behavior for up to an hour and twenty minutes. Samples of each on food and off food recordings are available (doi:10.5061/dryad.n9c88). Our camera hardware enables us to maintain an average frame capture rate of up to 30 Hz. In on-food sessions, video recording is truncated once animals have reached outside of the bacterial lawn. In off-food recordings, as animals reach close to the edge of the agar plate, the overall darkness of image frames increases and recording sessions were designed to stop automatically after a certain threshold. We concluded that a difference image algorithm used in our tracker is useful for recording *C. elegans* movement in a food-deprived environment.

Next, we assessed whether our image processing and image analysis algorithms can handle massive data obtained from the tracker. The performance of the segmentation process exceeds real time on our average workstation computers. As shown in Table 1, performance on individual Dell workstations are shown to average around 34 frames per second. However, with the use of multiple machines, long videos can be processed in a fraction of the time. The statistics in Table 1 are an average of the performance of each machine type across 42 video samples. Thirty eight machines were tested, giving 5 different hardware configurations; 4 Dell Vostro Desktops, 1 Dell T3700 Desktop, 1 Alienware Desktop, 2 Dell T1700 Desktops, and 30 Dell T7810 Desktops.

**Table 1:** Individual machine segmentation performance.

CPU	Clock	RAM	FPS	TPF (ms)
Intel Xeon	2.67 GHz	6 GB	27.9	35.9
Intel Core i7	2.80 GHz	8 GB	30.9	32.4
Intel Core i7	2.80 GHz	12 GB	31.7	31.6
Intel Xeon	2.40 GHz	16 GB	52.6	19.1
Intel Xeon	3.40 GHz	16 GB	54.9	18.2

The average performance in frames per second (FPS) and time per frame (TPF) for a variety of machines utilizing 4 threads.

In practice, our videos are processed on Dell T3610 Desktops (Xeon E5 @ 3.0GHz CPU and 8GB of RAM). These machines each have 6 physical cores and 6 additional logical cores, giving a capacity for 12 simultaneous threads. Performance in relation to available thread count is shown in Table 2. Although analysis can be conducted with the use of only one machine, each additional machine increases the performance of the software linearly. Our hardware configuration includes 32 desktops, capable of processing approximately 2,800 frames per second, or about a minute and a half of video per second.

**Table 2:** Multi-thread performance.

Threads	FPS
4	51.20
6	75.26
8	80.36
10	88.25
12	96.55

Performance scaling in relation to the number of threads available to the program.

With regard to the reliability of image processing, our methodology successfully segments and extracts centroid data in 98% (on average) of all frames with worms. The process tends to fail on frames that were recorded during camera movements, which are blurry, and frames in which the worm body is against the edge of the plate. In these cases, the data from the segmentation process is considered unreliable and is discarded.

### Extraction of Movement Features Reveals Different Behaviors on and off Food

Based on the camera position and centroid, we calculated speed and acceleration of animals; to visualize the results, we created a web user interface (UI) for selecting observation samples and viewing them as time series (http://medixsrv.cstcis.cti.depaul.edu/nematodes/visualizer/). The UI was designed to allow selection of multiple samples, enabling us to easily view and compare different animals side-by-side. The web UI has access to all available data samples, and can be accessed from anywhere without need for special software.

**Fig 5:**
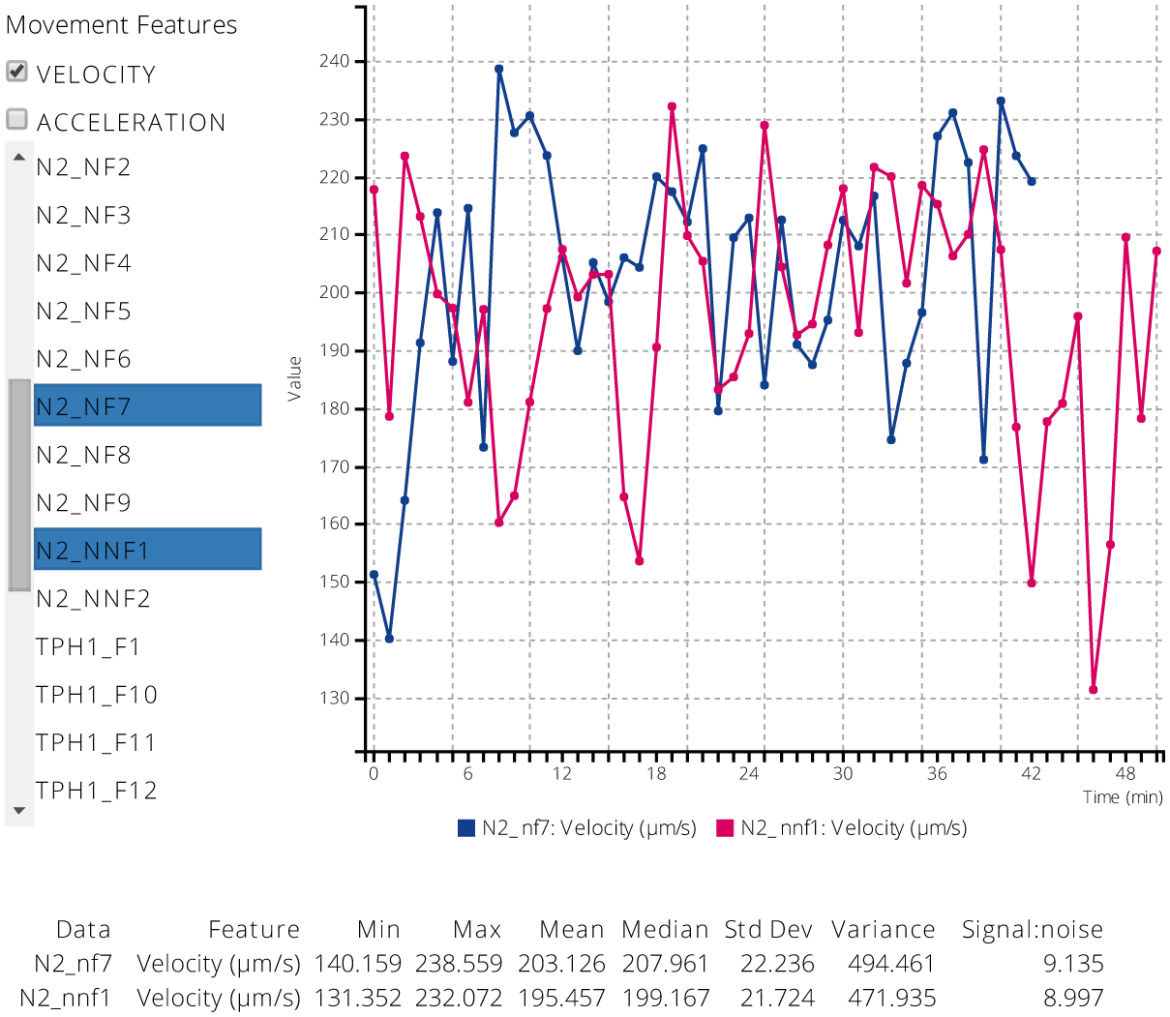
Movement feature web visualizer. Web user interface for visualizing movement feature time series on multiple observation samples; F, NF, and NNF stand for on-food, off-food, and pre-conditioned on a food-deprived agar plate for two hours before re-introduction to another plate without food, respectively.

When well-fed wild-type and *tph-1* animals were introduced to an agar plate without food, they exhibited consistently high speed movement for more than an hour (Fig 5). In fact, even if wild-type animals pre-conditioned on an agar plate lacking food for two hours were re-introduced to another plate without food, they showed consistently high speed. By contrast, when well-fed wild-type and *tph-1* animals were introduced to plates with abundant food, they showed different locomotory behaviors (Fig 5). As previously noted, *tph-1* mutants did not slow down over recording time. On the other hand, wild-type animals slowed down after 20 minutes of extended movement. Although we expected that *tph-1* would show a deficit in slowing down on food, it was surprising that the reduction of wild-type animal speed took more than 20 minutes.

### Path Structure Analysis Can Lead to a New Insight into Locomotory Behavior

**Fig 6:**
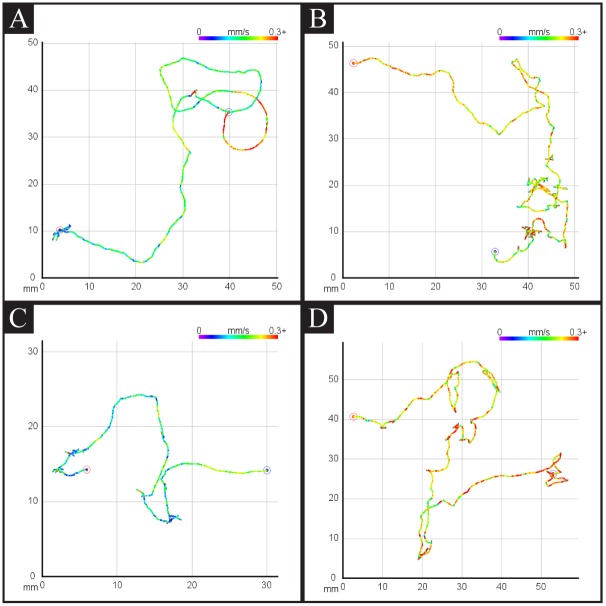
Representative centroid path from wild-type and mutant animals. Centroid movement path visualization. Color indicates the speed of the worm at that position. A: wild-type N2 on food, B: wild-type N2 off food, C: *tph-1* mutant on food, D: *tph-1* mutant off food.

Previous studies described how *C. elegans* locomotory behavior changes over time in response to removal of bacterial food. These studies were performed by measuring the frequency of movement parameters, such as turns and reversals, at intervals of tens of minutes. We intended to quantify food search behavior using path-based algorithms, which provide a holistic view of food search behavior. To visualize the movement path, we took two approaches: an animation, and a plotted path. The animation is a recreation of the worm path over time that plots the global centroid positions at a rate faster than real time. The points are persistent on the graph, thus producing a trail as the worm moves, and by the end, tracing the entire path of the worm. The plotted path is simply the last frame of this animation (Fig 6). Additionally, to read the position of the worm in the physical space, the physical space was divided into a grid of 1 mm square cells. Information about the worm’s speed and acceleration at each frame can also be included in these visualizations by coloring each centroid position as an indication of its speed at that time. Our visual inspection of the path structure indicates that when wild-type animals are freshly removed from food, their trajectories show a cluster in one area, then relatively large trajectories, followed by a cluster in another area (Fig 6B and 6D). However, this pattern disappears over time.

**Fig 7:**
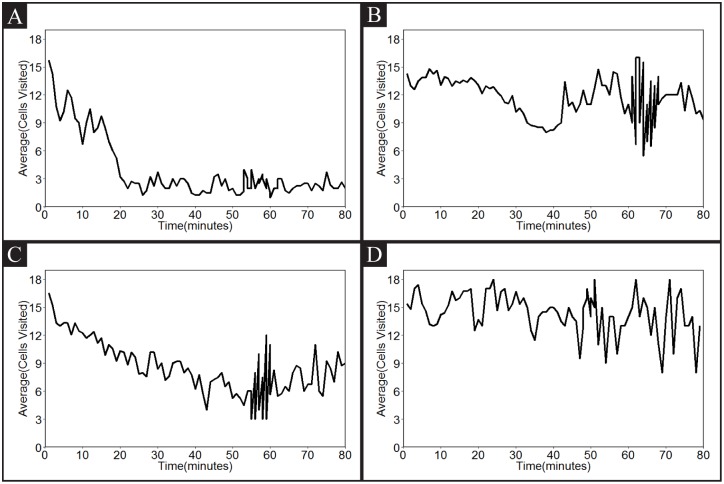
The number of cells visited over time. The Y axis represents the number of unique 1 mm^2^ cells the worm has visited during the 1 minute interval, the X axis is time in minutes. A: wild-type N2 on food (n = 4), B: wild-type N2 off food (n = 33), C: *tph-1* mutant on food (n = 15), D: *tph-1* mutant off food (n = 5).

We further analyzed their path structure by counting the number of grid cells the worm visited over time. As shown in Fig 7A and 7B, we made a comparison between N2 on-food and N2 off-food. We noticed that N2 on-food animals tend to visit many cells at the beginning, but much less frequently after approximately 20 minutes. However, N2 off-food animals maintain a higher number of cell visits throughout the observation period. Intriguingly, the number of cell visits has a correlation with the speed of animals over time. This behavioral difference in on- and off-food wild-type animals may result from serotonergic signaling, which is increased by food presence. Therefore, we also examined whether *tph-1* mutants, which have a defect in serotonin production, exhibit any difference from wild-type animals on- and off-food (Fig 7C and 7D). We found that *tph-1* mutants either on- or off-food behave like wild-type off-food animals, indicating that the suppression of wild-type animals’ speed is caused by serotonergic signaling.

### Wild-type Animals Can Exhibit the Lévy Flight Pattern in Search Space That Lacks Sensory Cues

The characteristic path structure of wild-type animals freshly removed from food led us to analyze their movement patterns with step lengths. A step length is defined as a distance between two centroids, each of which is associated with directional change. Our previous body curvature analysis showed that the head angles of wild-type animals moving in a straight direction fluctuate between -30 and 30 degrees [27]. Thus, we chose 40 degrees as the cut-off angle for directional change.

**Fig 8:**
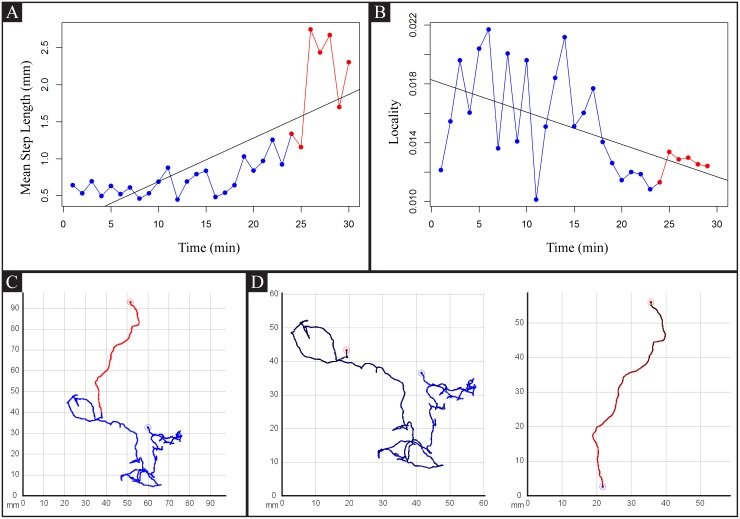
Step length, locality, and path. A: Mean step length over time for a wild-type N2 off food showcasing two distinct movement phases using different colors: the first phase (red) corresponds with “local” movement behavior with short step lengths. The second (blue) movement phase correspond with “global” movement behavior. B: Locality ($\frac{E[v]}{O}$) through time. When step length is low, the locality is usually high and vice versa. C: A complete path of the observation period (colored corresponding to the phases described in A). D: Zoomed path sections corresponding to the phases described in A.

**Fig 9:**
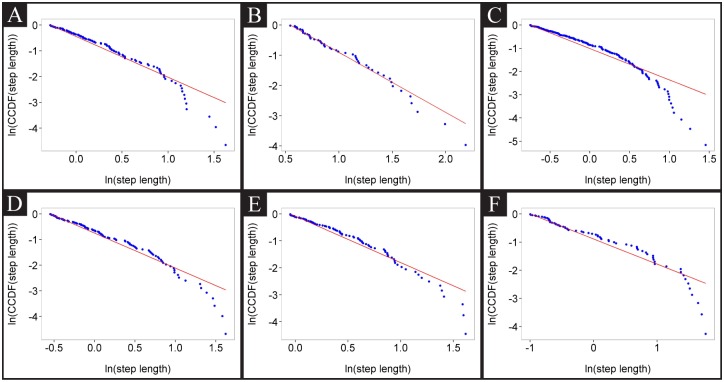
Empirical complement cumulative density functions (CCDF) vs maximum likelihood approximations. A-F: CCDF vs maximum likelihood approximations for the recordings labeled in Table 3.

The frequency of turns or reversals will be inversely correlated with the size of step lengths; if worms make frequent directional changes, then step lengths get shorter. In addition to information on the frequency of turning events, step length analysis provides information on the relocation of animals. To calculate how far animals displace from their previous spot, we measured locality, which inversely correlates with visited cell number but correlates with mean speed. Locality index is usually inversely correlated to step length size (Fig 8). Another intriguing observation was that wild-type animals exhibited random, small movement steps that were interrupted by large rare relocation steps when they were transferred to a bacteria-free agar plate. This food search pattern is described as Lévy flight in the literature, in which the rank distribution of movement step lengths is fit by a power-law distribution. Lévy flight is widely accepted as an optimal search strategy in environments where sensory information that provides target location is not present [17]. In order to determine that our observations are consistent with the hypothesis that the step length data are fit by a Lévy power-law distribution, we estimated the parameters of the power-law distribution function (Eq (8)) using the approaches described in the methodology section (Eqs (9) and (10)). A power-law exponent between 1 and 3 represents Lévy flights, whereas an exponent above 3 represents Brownian walks [28]. Table 3 summarizes the estimations of the parameters for step length distribution for each of the 33 sets of video data we recorded for wild-type N2 off-food. These animals were cultured on various densities of OP50 *E. coli* without any consideration that bacterial lawn thickness and uniformity may affect off-food locomotory behavior. Our results show that 6 out of the 33 videos for off-food N2 tracking can be fit by Lévy flight distribution (for angle 40 degrees and first 20 minutes), whereas the rest can be quantified with Brownian walks. Fig 9 shows logarithmic step length data that present a Lévy flight behavior. These data indicate that *C. elegans* can use Lévy flights as well as Brownian walks to locate food in a search space that lacks sensory cues.

**Table 3:** Estimated power law parameters from step length using Eqs (7)–(9).

	Length (min)	N step	Min step	Max step	Est. Xmin	N tail	Est. alpha	Distance
	12	213	0.009	13.339	2.113	19	3.600	0.920
	20	331	0.003	6.660	1.320	68	3.622	0.798
	8	106	0.043	7.289	2.516	16	4.260	0.858
	10	340	0.012	2.663	1.186	30	4.988	0.915
	20	364	0.002	4.136	1.482	42	4.491	0.887
	17	219	0.007	7.156	1.283	77	3.118	0.653
A	18	253	0.003	5.821	0.756	105	2.581	0.589
	20	445	0.002	5.825	1.038	90	3.509	0.802
	20	349	0.005	4.406	2.012	33	4.749	0.908
	17	306	0.002	6.267	1.243	54	3.034	0.827
	20	250	0.015	8.080	1.591	50	3.574	0.804
	12	265	0.002	4.002	1.714	28	5.456	0.898
	18	231	0.004	11.206	1.816	41	3.251	0.827
B	20	239	0.008	12.621	1.724	53	2.987	0.782
	20	317	0.005	5.215	1.578	47	4.143	0.855
	20	351	0.002	4.052	1.124	55	3.968	0.846
C	20	356	0.000	4.284	0.473	175	2.352	0.511
	20	380	0.000	3.144	1.433	36	5.781	0.908
	20	345	0.000	7.446	1.789	26	4.486	0.928
	20	345	0.001	3.743	1.525	40	4.860	0.887
	20	276	0.016	10.598	1.530	75	3.502	0.732
	20	384	0.000	3.630	0.884	84	3.126	0.784
	12	199	0.004	4.091	1.231	65	3.259	0.678
	16	222	0.022	4.383	1.741	24	5.449	0.896
	20	360	0.007	3.257	1.590	26	6.126	0.931
	20	331	0.005	3.866	1.801	39	4.922	0.885
	20	319	0.001	6.337	1.671	53	3.883	0.837
D	20	326	0.000	5.397	0.578	108	2.366	0.672
E	19	265	0.004	5.314	0.953	86	2.722	0.683
	20	258	0.014	5.929	2.956	17	6.938	0.938
	20	335	0.001	4.062	1.655	41	5.875	0.881
	20	656	0.002	2.915	1.008	64	4.608	0.904
F	9	116	0.018	7.607	0.369	71	1.893	0.405

Our results show that 6 out of the 33 videos for wild-type off-food data can be quantified with Lévy flight distribution (for angle 40 degrees and first 20 minutes). Fitting of the six indicated data sets (A-F) to power-law distributions is shown in Fig 9. The column labels have the following meaning: N step = total number of steps for that video. Estimated Xmin = minimum step length from which the distribution to the right follows a Lévy flight distribution. N tail = number of steps that have a length greater than estimated Xmin. Distance = distance between the power-law distribution and the empirical distribution. By definition, the value of alpha between 1 and 3 fits to Lévy flights [29]. The values below 1 and above 3 resemble ballistic and Brownian movements, respectively [28].

### An Example of *tph-1* Mutant Step Lengths is Consistent with a Worm that Exhibits Continuous Searching Behavior on Food

**Fig 10:**
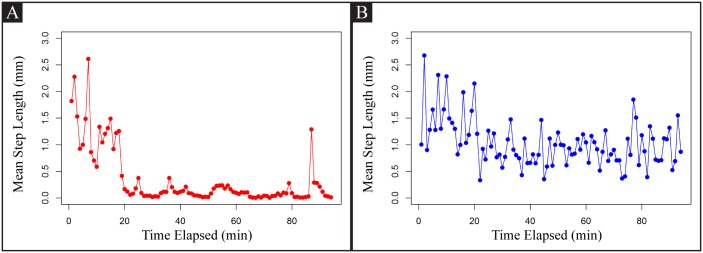
Step length vs time. Mean step length over time. A: N2 on food. B: *tph-1* on food.

*tph-1* mutants exhibit several food-associated deficits. Our initial assessment of *tph-1* mutants off-food showed that their search behavior was not so different from that of wild-type off-food animals. We further examined whether *tph-1* mutants exhibited any deficits on food using step length analysis (Fig 10). A wild-type animal on food initially exhibited exploratory behavior, which was associated with large step lengths. However, after 15-20 min on food, its step lengths were reduced to a minimum level. Wild-type animals in long-term recordings showed occasional large step lengths, which were observed in roaming animals. By contrast, a *tph-1* mutant consistently showed a mixture of large and small step lengths. This characteristic has some resemblance to food search behavior of off-food animals. Because *tph-1* mutants on food do not make frequent turns and reversals as often as wild-type animals freshly exposed to a food-deprived environment [30], these results strongly suggest that *tph-1* animals on food resemble later stages of food-deprived wild-type animals engaging global search.

## Discussion and Conclusion

A primary aim of our tracker was to record the movements of a single worm in the absence of food over a long period of time. A food-deficient environment poses a challenge for tracking worm movement, since food-deprived worms move faster with frequent turns and tend to reach the edge of the agar plate more quickly than worms on a bacterial lawn. Previously described worm trackers, which were developed for different purposes, are not optimal for our aim. For example, Luo et al. developed a tracking system that uses a high-pixel density camera to record the movements of multiple young adult worms performing salt chemotaxis across 25 cm x 25 cm agar plates [31]. Data analysis was performed using customized particle-tracking and shape analysis algorithms written in two proprietary software packages. While this system is robust, it is difficult to acquire images at a high frame rate and to analyze the detailed curvature of individual animals. Another system has a similar set up as ours in respect to hardware logistics [9]; the position information of a single animal is fed to the controller that readjusts the position of the motorized stage, in which a camera is mounted. This system has been successfully used for tracking single animals on food, but is not robust enough to track single animals in the absence of food. To reliably record worm movement in the absence of food, we changed the latter tracker in two different aspects. First, we used a new algorithm to track worm movement. One of the frequent issues in tracking worms is that tracking software recognizes air bubbles or precipitates resulting from NGM agar plate preparation as a worm. We applied a difference image algorithm, in which an image frame is subtracted from another previous image frame, canceling out all of the background pixels and leaving only a worm image. We find that our new algorithm reliably distinguishes a worm from salt precipitates or other blobs. Second, we modified the housing environment to accommodate a 150 mm diameter NGM agar plate which is comparatively larger than conventional 60-100 mm diameter NGM plates used in other worm trackers [11, 13, 32, 33]. With a 60 mm plate, we can only record less than 2 minutes, as animals, upon food deprivation, tend to reach the edge rapidly. With a 150 mm plate, we typically record more than 15 min, and occasionally, more than an hour. Together, the changes we implemented in our worm tracking system significantly improved off-food worm recording.

Another goal of our tracker was to identify novel metrics by which we could parameterize or quantify worm behavior. We implemented algorithms described in other published worm trackers [11, 19, 20, 32], to satisfy essential functions of worm tracking, such as image processing and segmentation, and the extraction of commonly used features, such as movement features and centroid location. The current version of our tracker allows the quantification of these metrics, as well as introduces novel metrics that provide insights into food search behavior. In particular, we implemented several metrics for analyzing path structure, such as cell occupancy, step length and locality. The cell occupancy metric divides the physical space into defined areas, which we consider “searched” if the worm is ever present within its boundaries. This gives us a rough estimate of the amount of space the worm was able to search within the observation period. Although the methods used in Humphries et al. [22] work with simulation data, we suspect that many of the theories can be applied to *C. elegans* behavioral data. Step length analysis gives a unique perspective in path analysis and can replace previous movement-based analysis of locomotory behavior, such as turns and reversals, when overall worm path structures are considered. Locality can be used as a complementary metric to step length analysis, since step length analysis does not give information on how much the worm is displaced in two dimensional spaces from a previous measurement point. Based on our preliminary results for predictive modeling of movement, we plan to integrate an auto-correlation analysis of the step length data and angles using approaches proposed by Reynolds A. [25] and Dray et al. [34]

Previous studies found that upon exposure to a food-deprived environment, well-fed *C. elegans* exhibits restricted area search/local search or dispersal/global search depending on exposure duration [4, 14, 15]. These studies relied on the frequencies of turns and reversals, which are movement parameters rather than genuine path-based parameters. In our current study, we determined the path of animals using step lengths and search efficiency by analyzing long continuous video frames. Intriguingly, the local/global search behavior overlaps with another movement pattern, which has some similarities to Lévy flights. In Lévy flights, a cluster of small random movements is interspersed by infrequent longer relocations [22, 35]. Lévy flights reduce oversampling compared with Brownian walks. In other words, animals using the Lévy flight strategy are less likely to return to previously searched areas due to the long relocations. For this reason, Lévy flights, but not Brownian walks, have been postulated to be the optimal search strategy in an unknown environment. In fact, Lévy search strategy has been observed in many species, including *Drosophila* [36], mussels [37], birds [38, 39], marine predators [29], and humans [40]. Peliti et al. [41] showed that worms exposed to a food-deprived environment exhibit directional movement. If worm movements in the absence of food are purely random Brownian walks, then such directional locomotion could not be observed. Thus, these results support our finding that initial *C. elegans* food search behavior combines random walks with long relocations.

Our analysis of step length distribution using the maximum-likelihood estimate method indicates that a fraction of the samples (6 out of the 33 off-food animals) fits into the mathematical definition of Lévy flight. The remaining samples fit into Brownian, or possibly complex, composite movements. Hence, *C. elegans* can adopt different search patterns on an individual basis. Such variability in search patterns was also observed in the *C. elegans* local/global search paradigm. The exact timing of the transition from local to global search in animals exposed to a food-free environment varies from 10 min to 45 min [14]. Furthermore, the transition could be abrupt or gradual. In some cases, such a transition was not observed. How can we reconcile behavioral variability in a genetically homogeneous population? A recent study showed that sensory experience on food affects subsequent off-food behavior [42]. Specifically, if animals were cultured in a highly variable density of bacterial food, they made less frequent turns. Because we did not control bacterial thickness and uniformity, to which animals were exposed during their growth, different culture conditions might explain why *C. elegans* exhibited Lévy flights or Brownian walks, or both. The variability in movement patterns was also observed in albatrosses and marine predators [17, 29]. Similar to *C. elegans*, these animals exhibited Lévy flights, Brownian, or complex composite movements. This variability was attributed to different environments such as food abundance.

*tph-1* mutants, which fail to produce serotonin, exhibit many behavioral and metabolic changes that are associated with food [30, 43–46]. *tph-1* mutants have a defect in slowing when they encounter food [47]. In addition, *tph-1* mutants exhibit an enhanced roaming response on food. Together, these studies implicated serotonin as a neuromodulator for locomotory behavior. Our observation of *tph-1* suggests that *tph-1* mutants continue food search behavior even on food. In the future, it will be necessary to analyze other mutants that have a defect in serotonin signaling, including serotonin receptors (*mod-1*, *ser-1* to *ser-7*) and transporters (*cat-1*, *mod-5*) to confirm our findings.

In summary, we have developed methods for recording and tracking *C. elegans* over long periods of time, as well as algorithms for extracting information about its locomotory behavior. In our study, we employed these methods in an attempt to identify differences in food-related behavior between wild-type and *tph-1* animals. Our study illustrates an example of how our quantitative assessment system can be used to study the genetic basis of behavior. In the future, it will be possible to expand our study to analyze other mutants that have defects in food sensing or food-related behaviors. Alternatively, it will be possible to examine transgenic animals whose neural circuits that potentially control food searching have been modified.

## Supporting Information

S1 Fig Path Graphs for the 6 Videos Presented in Fig 9. (EPS)

S2 Fig Mean step length vs. reversals. A: Mean step length of a *tph-1* mutant on food. B: Reversals of the same *tph-1* mutant on food. The size of the mean step length shows an inversed relationship with the frequency of reversals over a period of time.
(EPS)
